# Supplementary material for: DisiMiR: Predicting Pathogenic miRNAs Using Network Influence and miRNA Conservation
Source: Noncoding RNA. 2022 Jun 23;8(4):45. doi: 10.3390/ncrna8040045 (PMC9326518; doi:10.3390/ncrna8040045)
Supplement: Supplementary file 1 [file ncrna-08-00045-s001.zip › ncrna-1746534-SI.pdf]

DisiMiR: Predicting Pathogenic miRNAs using Network Influence and miRNA Conservation

Kevin R. Wang[1]* and Michael J. McGeachie[2]

[1] Roxbury Latin, Boston, MA, 02132, USA
[2] Channing Division of Network Medicine, Brigham and Women's Hospital and Harvard Medical School, Boston, MA, 02115, USA

Supplemental Material

Supplemental Data Files

alzheimers_false_pos.csv
breast_cancer_false_pos.csv
gastric_false_pos.csv
hepato_false_pos.csv

These four supplemental data files document the literature verification of the miRs identified as causal by DisiMiR, but which were not causal according to the HMDD database. These false-positives constitute hypotheses: the miRNA in question may be causally associated with the disease in question, but either there has not been sufficient research to establish this relationship, or such research was published after the HMDD was curated. Recent literature was retrieved using the PubMED Entrez API by combining the miR name with the relevant disease. If the query returned no papers, it was removed from consideration. If the abstract or the manuscript demonstrated that the miRNA played a causal role in the given disease, the hypothesis was marked as pathogenic and the PubMED identifiers (PMIDs) for the relevant papers was provided. All miRNA queries were performed on 6/10/22.

Each of these four files has the same structure. The miRNAs column shows the specific miR that was used in the search. The Manual Check column lists PMIDs of papers indicating that the given miRNA played a causal role in the given disease. It is important to note that this column does not contain all of the papers that indicated a causal role, only the first few. The Pathogenicity column indicates if the miRNA was found to be causal. The num_papers_returned column shows the total number of papers returned by the PubMED query. The Causal_Papers and Non_Causal_Papers columns show the PMIDs of papers judged to present causal evidence, or not.

Supplementary Figure S1

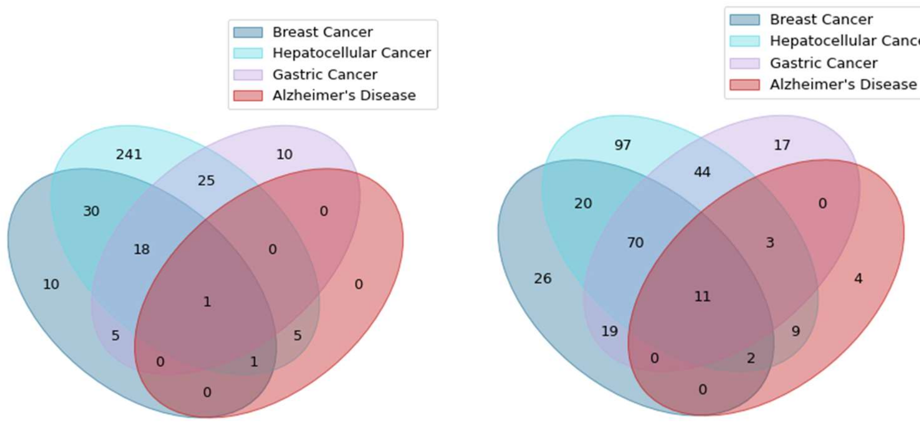

DisiMiR Causal Predictions Overlap   HMDD Causal Associations Overlap

Supplementary Figure S1: Venn diagrams showing the overlap in causal miRNAs for each of the four diseases we examined, considering the 758 miRNAs that were present in all four miRNA expression datasets. LEFT: DisiMiR's predictions of causal miRNAs. RIGHT: HMDD listing of causal miRNAs.

Supplementary Table S1

Supplementary Table S1: DisiMiR accuracy with different thresholds. By using a different threshold for adjudicating causal and not-causal miRNAs, DisiMiR makes gains in sensitivity with little losses in its specificity.

Disease	Disease Causal Accuracy				Sensitivity	Specificity
	True Negative	False Positive	False Negative	True Positive		
Breast Cancer	564	113	51	100	0.662	0.833
Alzheimer's Disease	2465	16	28	12	0.300	0.994
Hepatocellular Cancer	1971	203	47	348	0.881	0.907
Gastric Cancer	2090	164	105	165	0.611	0.927
